# Leptospirosis Outbreak in Aftermath of Hurricane Fiona — Puerto Rico, 2022

**DOI:** 10.15585/mmwr.mm7335a2

**Published:** 2024-09-05

**Authors:** Forrest K. Jones, Abigail G. Medina, Kyle R. Ryff, Jessica Irizarry-Ramos, Joshua M. Wong, Eduardo O’Neill, Ismael A. Rodríguez, Iris Cardona, Lorena Hernández, Alfonso C. Hernandez-Romieu, Maile T. Phillips, Michael A. Johansson, Tesfaye Bayleyegn, Christine Atherstone, Katherine Roguski DeBord, María E. Negrón, Renee Galloway, Laura E. Adams, Melissa Marzán-Rodríguez

**Affiliations:** ^1^Epidemic Intelligence Service, CDC; ^2^Division of Epidemiology, Puerto Rico Department of Health; ^3^Office of Island Affairs, National Center for State, Tribal, Local, and Territorial Public Health Infrastructure and Workforce, CDC; ^4^Division of Vector-Borne Diseases, National Center for Emerging and Zoonotic Infectious Diseases, CDC; ^5^Public Health Laboratory Institute, Puerto Rico Department of Health; ^6^Division of Environmental Health Science and Practice, National Center for Environmental Health, CDC; ^7^Division of High-Consequence Pathogens and Pathology, National Center for Emerging and Zoonotic Infectious Diseases, CDC.

**Summary**

**What is already known about this topic?**

Leptospirosis, an acute bacterial zoonotic disease, can progress to severe, potentially fatal illness. Increased incidence has been associated with flooding in areas around the world where the disease is endemic.

**What is added by this report?**

In 2022, a large leptospirosis outbreak occurred in Puerto Rico after Hurricane Fiona made landfall. Proactive public health response activities leveraged existing surveillance and laboratory capacity. The increase in reported cases was likely the result of a combination of widespread exposure to contaminated water and increased testing.

**What are the implications for public health practice?**

Robust laboratory and epidemiologic surveillance combined with outreach to health care providers after flooding events can improve leptospirosis case identification, inform clinicians considering early initiation of antibiotic therapy, and guide public messaging to avoid contact with floodwaters.

## Abstract

Leptospirosis, an acute bacterial zoonotic disease, is endemic in Puerto Rico. Infection in approximately 10%–15% of patients with clinical disease progresses to severe, potentially fatal illness. Increased incidence has been associated with flooding in endemic areas around the world. In 2022, Hurricane Fiona, a Category 1 hurricane, made landfall and inundated Puerto Rico with heavy rainfall and severe flooding, increasing the risk for a leptospirosis outbreak. In response, the Puerto Rico Department of Health (PRDH) changed guidelines to make leptospirosis cases reportable within 24 hours, centralized the case investigation management system, and provided training and messaging to health care providers. To evaluate changes in risk for leptospirosis after Hurricane Fiona to that before the storm, the increase in cases was quantified, and patient characteristics and geographic distribution were compared. During the 15 weeks after Hurricane Fiona, 156 patients experienced signs and symptoms of leptospirosis and had a specimen with a positive laboratory result reported to PRDH. The mean weekly number of cases during this period was 10.4, which is 3.6 as high as the weekly number of cases during the previous 37 weeks (2.9). After Hurricane Fiona, the proportion of cases indicating exposure to potentially contaminated water increased from 11% to 35%, and the number of persons receiving testing increased; these factors likely led to the resulting overall surge in reported cases. Robust surveillance combined with outreach to health care providers after flooding events can improve leptospirosis case identification, inform clinicians considering early initiation of treatment, and guide public messaging to avoid wading, swimming, or any contact with potentially contaminated floodwaters.

## Introduction

Leptospirosis, an acute bacterial zoonotic disease, is endemic in Puerto Rico, which reports higher numbers of annual leptospirosis cases than any other U.S. jurisdiction (*1*–*4*). Previous hurricanes in Puerto Rico have been followed by increased leptospirosis incidence (*5*,*6*). Pathogenic *Leptospira* bacteria (the causative agent) can survive in soil and water, are maintained in animal hosts, and are transmitted through the urine of infected animals. Leptospirosis infection in humans causes a spectrum of disease severity. Most illness is mild and characterized by fever, chills, myalgia, nausea, vomiting, diarrhea, headache, conjunctivitis, and other signs and symptoms. Infection in approximately 10%–15% of patients with clinical disease progresses to severe, potentially fatal illness with multiorgan involvement that can include renal failure, liver failure, pulmonary hemorrhage, and meningitis (*7*,*8*).

Leptospirosis can be challenging to diagnose because infection can cause a wide range of nonspecific symptoms, clinical presentation can be confused with other diseases, and the sensitivity and specificity of the laboratory diagnostics depend on when a sample is collected: real-time polymerase chain reaction (PCR) testing is recommended when the bacteria are most likely to be present in blood (approximately 4–6 days post-illness onset); in contrast, serologic tests have low sensitivity in the first week after illness onset given the time for the immune response to generate antibodies (3–10 days after symptom onset) (*9*). A negative real-time PCR or serologic test result from a specimen collected in the acute phase of illness does not rule out infection. Patients who have received only negative test results from specimens collected during the first week of illness are recommended to have serologic testing of a convalescent sample collected 7–14 days after the first sample. However, collection of specimens during convalescence is challenging because it requires patients to return for repeat testing. Among patients clinically suspected to have leptospirosis, initiation of empiric antibiotic treatment (e.g., doxycycline) is recommended while awaiting laboratory results (*9*). Early recognition and treatment with antibiotics for patients in whom leptospirosis is suspected reduces morbidity and mortality.

## Epidemiologic Investigation and Results

Because of an expected increase in leptospirosis cases after heavy flooding when Category 1 Hurricane Fiona made landfall on September 18, 2022, PRDH took action to strengthen surveillance, guide response efforts, and improve patient outcomes. Leptospirosis cases were identified through the existing passive surveillance system. This includes reporting by health care providers of patients with observed clinically compatible illness as suspected cases and laboratories (including hospitals, private laboratories, and the PRDH laboratory) reporting results for specimens submitted for leptospirosis testing. A confirmed case was defined as a suspected case with a positive real-time PCR result for leptospirosis, and a probable case was defined as a suspected case with detection of leptospirosis-specific immunoglobulin M (IgM) antibodies by enzyme linked immunosorbent assay (ELISA).* Microagglutination testing, the reference standard serologic test for leptospirosis, was considered for probable cases but was not possible because of shipping difficulties. For each case, all available test results were used for case classification (Supplementary Figure, https://stacks.cdc.gov/view/cdc/160382), including those from both acute- and convalescent-phase specimens (those collected ≤7 days and >7 days after symptom onset, respectively).

To evaluate differences in confirmed and probable cases before and after Hurricane Fiona, leptospirosis cases with onset dates during the 37 weeks before Hurricane Fiona (January 2–September 17, 2022) were compared with cases with onset dates during the 15 weeks after Hurricane Fiona (September 18–December 31, 2022). The pre-hurricane period of 37 weeks was chosen to characterize the cases before Hurricane Fiona and to obtain the number of cases sufficient for statistical power; the post-hurricane period of 15 weeks was chosen for evaluation because the number of cases had generally stopped decreasing and began to plateau at this time. To estimate the relative increase in cases, the mean weekly number of confirmed and probable cases before and after Hurricane Fiona were compared. Annual municipality-level incidences of confirmed and probable cases during both periods were calculated by dividing the number of cases by the population size (from the 2020 U.S. Census) and duration of the period (i.e., 37 weeks before Hurricane Fiona and 15 weeks after Hurricane Fiona), and then multiplying by 52 weeks.

To investigate whether persons who received a positive leptospirosis test result might have been infected with dengue virus, which causes similar signs and symptoms and is endemic in Puerto Rico, the PRDH Passive Arboviral Disease Surveillance System was searched for dengue cases among persons who had the exact same date of birth, similar patient name, and date of symptom onset within 2 weeks of the identified leptospirosis cases. Leptospirosis case trends during January 3, 2021–September 30, 2023 were also compared.^†^ This activity was reviewed by CDC, deemed not research, and was conducted consistent with applicable federal law and CDC policy.^§^

During the 15 weeks after Hurricane Fiona made landfall, 823 suspected leptospirosis cases were reported to PRDH: 91 with only real-time PCR test results, 157 with only IgM ELISA results, 573 with results from both tests, and two without results from either tests; 156 of the 823 suspected cases were categorized as either confirmed from a positive real-time PCR test result (40, 26%) or as probable by IgM ELISA (116, 74%) (Table). The median age of persons with confirmed and probable cases was 42 years (IQR = 29–60 years); 116 (74%) were male. Among confirmed and probable cases, the most frequently reported symptoms were fever (83%), headache (65%), myalgia (56%), abdominal pain (47%), nausea (44%), and diarrhea (42%). A total of 112 (72%) patients were hospitalized and 10 (6%) died. Overall, 148 (95%) patients received antibiotic treatment. Sixteen persons (10%) with probable cases also received a positive test result for dengue virus infection by reverse transcription–polymerase chain reaction (RT-PCR).

**TABLE:** Description of confirmed and probable leptospirosis cases before and after Hurricane Fiona* — Puerto Rico, 2022

Characteristic	No. (%)		p-value^†^
	37 weeks before Hurricane Fiona landfall n = 108	15 weeks after Hurricane Fiona landfall n = 156	
**Case classification**			
Confirmed	16 (15)	40 (26)	0.034
Probable	92 (85)	116 (74)	
**Median age, yrs (IQR)**	48 (38–59)	42 (29–60)	0.037
**Sex**			
Female	27 (25)	40 (26)	>0.9
Male	81 (75)	116 (74)	
**Leptospirosis testing**			
Both IgM and real-time PCR testing	48 (44)	113 (72)	<0.001
IgM testing only	58 (54)	34 (22)	
Real-time PCR testing only	2 (2)	9 (6)	
**Outcome**			
Hospitalized	100 (93)	112 (72)	<0.001
Died	15 (14)	10 (6)	0.041
**Received antibiotic treatment** ^§^	107 (99)	148 (95)	0.087
**Contact source**			
Potentially contaminated water^¶^	12 (11)	55 (35)	<0.001
Potentially contaminated food^¶^	17 (16)	29 (19)	0.5
Animals (including pets)^¶^	66 (61)	100 (64)	0.6
Had occupational risk factor**	16 (15)	29 (19)	0.4
**Dengue test results**			
No testing performed	85 (79)	104 (67)	0.054
RT-PCR– or IgM-negative	19 (18)	36 (23)	
RT-PCR–positive	4 (4)	16 (10)	
**Sign or symptom**			
Fever^††^	69 (64)	129 (83)	<0.001
Myalgia^††^	57 (53)	88 (56)	0.6
Headache^††^	53 (49)	102 (65)	0.008
Conjunctivitis^††^	11 (10)	13 (8)	0.6
Thrombocytopenia^††^	36 (33)	53 (34)	>0.9
Rash^††^	11 (10)	45 (29)	<0.001
Persistent vomiting^††^	31 (29)	48 (31)	0.7
Abdominal pain^††^	57 (53)	73 (47)	0.3
Severe bleeding^††^	15 (14)	22 (14)	>0.9
Nausea^††^	43 (40)	69 (44)	0.5
Diarrhea^††^	46 (43)	66 (42)	>0.9
Kidney failure	23 (21)	29 (19)	0.6
Liver failure	11 (10)	14 (10)	0.7
Meningitis	1 (1)	1 (1)	>0.9

**Abbreviations:** IgM = immunoglobulin M; PCR = polymerase chain reaction; RT-PCR = reverse transcription PCR.

* Hurricane Fiona made landfall in Puerto Rico on September 18, 2022.

^†^ Pearson's chi-square test; Wilcoxon rank sum test; Fisher's exact test.

^§^ Antibiotic treatment status was missing for one person.

^¶^ Information on contact with potentially contaminated water, potentially contaminated food, or animals was missing for four persons.

** Included farmer, rancher, fishery sector worker, veterinarian, slaughterhouse worker, and animal caretaker, among others.

^††^ Information for the following signs and symptoms was missing for six persons: fever, myalgia, headache, conjunctivitis, thrombocytopenia, rash, persistent vomiting, abdominal pain, severe bleeding, nausea, and diarrhea.

**FIGURE 1:**
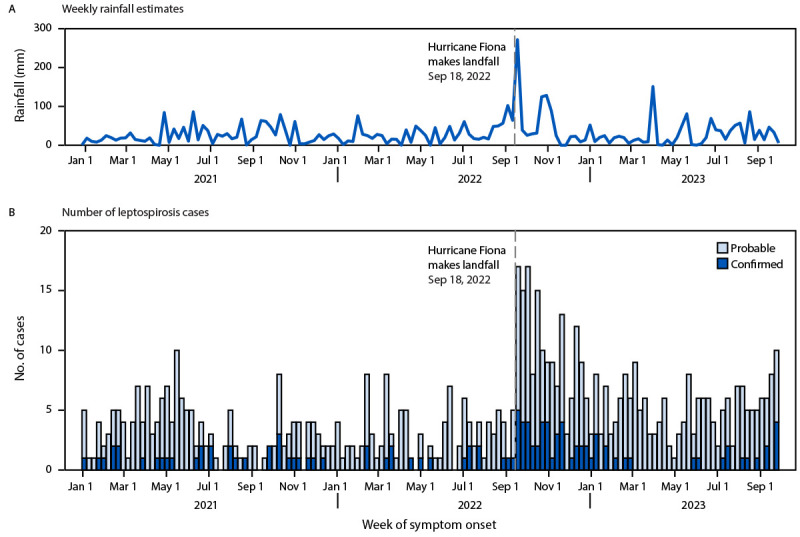
Weekly rainfall estimates* (A) and number of probable and confirmed leptospirosis cases (B) before and after Hurricane Fiona landfall — Puerto Rico, January 3, 2021–September 30, 2023 * https://climateserv.servirglobal.net/map

Incidence of confirmed and probable cases was highest during the first 5 weeks after Hurricane Fiona (14.4 per week) (Figure 1). The average weekly number of confirmed and probable cases during the 15 weeks after Hurricane Fiona (10.4) was 3.6 (95% CI = 2.6–4.9) times as high as that during the 37 weeks before landfall (2.9). The number of weekly confirmed and probable leptospirosis cases remained elevated throughout 2023: the average weekly number of cases was 3.3 in 2021, 5.1 in 2022, and 5.2 in 2023.

The proportion of patients with severe illness was lower during the 15 weeks after Hurricane Fiona than during the 37 weeks before: hospitalized (72% versus 93%, p<0.01) and died (6% versus 14%, p = 0.04) (Table). Patients with symptom onset after Hurricane Fiona more frequently reported exposure to potentially contaminated water than before the hurricane (35% versus 11%). The frequency of other exposures was similar before and after Hurricane Fiona: contact with animals (64% versus 61%), contact with potentially contaminated foods (19% versus 16%), and occupational exposures (e.g., farmer, rancher, fishery sector worker, veterinarian, slaughterhouse worker, or animal caretaker) (19% versus 15%).

**FIGURE 2:**
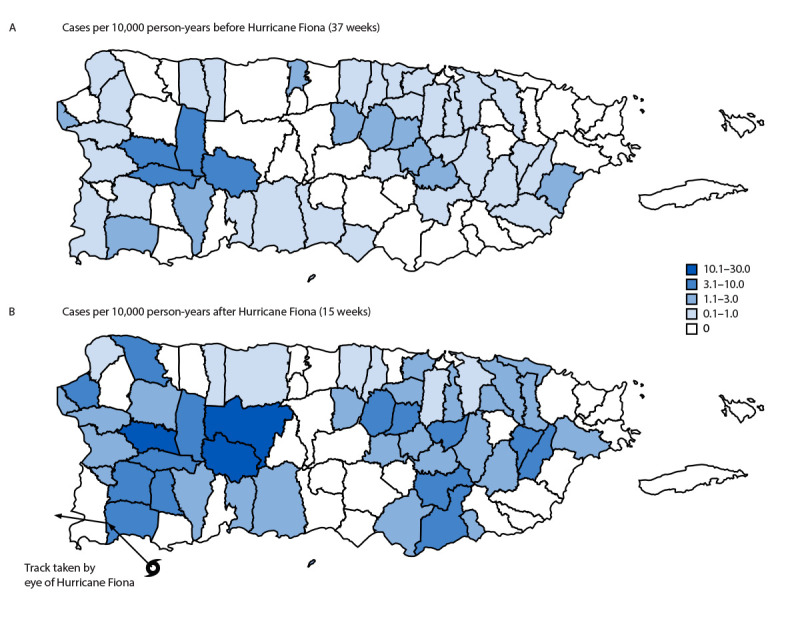
Municipality-level incidence of probable and confirmed leptospirosis cases* before (A) (N = 108) and after (B) (N = 156) Hurricane Fiona landfall — Puerto Rico, January 2–December 31, 2022 * Overall incidence of confirmed and probable cases (per 10,000 person-years) was 0.5 before Hurricane Fiona and 1.7 after Hurricane Fiona.

Patients with symptom onset after Hurricane Fiona resided in 48 (62%) of 78 municipalities, compared with patients with symptom onset before the hurricane, who resided in 43 (55%) of 78 municipalities (Figure 2). Thirty-three municipalities had one or more cases both before and after Hurricane Fiona; during both periods, the incidence was highest in the inland western municipalities.

## Public Health Response

Anticipating increased risk for leptospirosis from flooding, PRDH requested technical assistance to form a response team from CDC on September 20, 2 days after Hurricane Fiona made landfall. Objectives were to increase health care provider awareness, support laboratory testing, and facilitate reporting and analysis of leptospirosis cases. On September 23, 2022, the required provider reporting time was reduced from within 5 days to within 24 hours. Additional efforts to improve surveillance capacity included streamlining surveillance data collection and centralizing data entry into a single reporting system. On September 28, PRDH issued a leptospirosis clinical management and surveillance guide. A virtual clinical training (a video with on-demand access is available online^¶^) was created for health care providers to emphasize the importance of recognizing and reporting leptospirosis cases and early treatment with antibiotics like doxycycline to reduce severe disease and mortality. The training also discussed dengue case recognition, given the similarities between dengue and leptospirosis clinical presentations. Diagnostic capacity at the PRDH public health laboratory was strengthened by increasing the availability of reagents for PCR and IgM ELISA tests and designating additional PRDH staff members for sample processing and testing. PRDH continued proactive communication with the public through continuous messaging on social media about avoiding wading, swimming, or any contact with potentially contaminated floodwaters and providing weekly epidemiologic reports on the PRDH website.

## Discussion

A large increase in leptospirosis cases occurred immediately after Hurricane Fiona made landfall, with elevated case counts lasting >3 months. Rapid public health response efforts led to increased availability of surveillance data to monitor the outbreak as it evolved and provided timely, accurate information to the community about leptospirosis risk and prevention.

Increased exposure to pathogenic *Leptospira* bacteria from contaminated floodwater likely resulted in increased incidence of leptospirosis after Hurricane Fiona. This hypothesis is consistent with the increase in the proportion of patients who reported exposure to potentially contaminated water from 11% during the period before landfall to 35% after landfall. Increased physician awareness and testing likely also contributed to the increased reported incidence. Severe outcomes (hospitalization and death) were less frequent among persons with cases reported after Hurricane Fiona. Possible explanations for this decrease include increased detection of less severe cases, earlier initiation of treatment, or both. Weekly case numbers remained elevated through September 2023, which might reflect a sustained increase in case detection.

One primary challenge was the variability of leptospirosis diagnostic test sensitivity and specificity at different time points after a patient’s symptom onset. Although real-time PCR assays have low sensitivity >1 week after symptom onset, leptospirosis serologic tests have limited sensitivity during the first week after symptom onset because of the time until appearance of IgM antibodies (i.e., 3–10 days) (*9*). In addition, some probable cases might have had false positive IgM ELISA results, as reported in other areas with endemic disease, because of persistence of IgM antibodies from a previous infection for ≥12 months (*10*). In such cases, the symptoms relating to the person seeking care and testing might be caused by a different pathogen. Although coinfection could not be ruled out, the 16 probable cases of leptospirosis that had confirmed dengue infections by RT-PCR would be consistent with this scenario.

## Implications for Public Health Practice

Early case identification and treatment are critical to reducing morbidity and mortality associated with leptospirosis. In areas with endemic leptospirosis, health departments can reinforce leptospirosis surveillance and increase both public and clinician awareness, particularly during hurricane season or months with high risk for flooding. Maintaining and strengthening leptospirosis surveillance in Puerto Rico will help identify populations at risk, guide prevention and response recommendations, protect health, and better prepare for the impact of future hurricane or flooding events.

## Footnotes

* IgM tests conducted at the PRDH laboratory used the GenBio IgM ImmunoDOT *Leptospira* assay. IgM assay types used at private and hospital laboratories were not available in the data sets analyzed.

† Code used for analyses is available at https://github.com/fjones2222/lepto-hurricane-fiona.

§ 45 C.F.R. part 46.102(l) (2), 21 C.F.R. part 56; 42 U.S.C. Sect. 241(d); 5 U.S.C. Sect. 552a; 44 U.S.C. Sect. 3501 et seq.

¶
https://www.salud.pr.gov/CMS/493
